# Comparison of Reliability and Validity of the Chinese Four-Level and Three-District Triage Standard and the Australasian Triage Scale

**DOI:** 10.1155/2019/8490152

**Published:** 2019-11-14

**Authors:** Aiqun Zhu, Jingping Zhang, Huilin Zhang, Xiao Liu

**Affiliations:** ^1^Department of Nursing, The Second Xiangya Hospital of Central South University, Changsha, Hunan 410011, China; ^2^Emergency Department, The Second Xiangya Hospital of Central South University, Changsha, Hunan 410011, China; ^3^Nursing Psychology Research Center of Xiangya Nursing School, Central South University, Changsha, Hunan 410013, China

## Abstract

Emergency triage is an important tool for prioritizing urgent or critical patients, and its effect needs to be investigated and evaluated. This observational study aimed to compare the reliability and validity of the Chinese four-level and three-district triage standard (CHT) and the Australasian Triage Scale (ATS) in an adult emergency department of a general hospital in China. From 2016-01 to 2017-01, twelve nurses independently performed on-site triage of 254 patients and 1552 patients to assess the scales' reliability and validity, respectively. The interrater reliability, as assessed by the weighted *k* scores, was 0.686 (95% CI 0.608–0.757) for the CHT and 0.731 (95% CI 0.663–0.790) for the ATS, and the *k* scores between the CHT and the ATS were 0.630 (95% CI 0.594–0.669). Temperature, respiration, pulse, blood oxygen saturation, waiting time, treatment time, emergency disposition, hospitalization rate, and mortality were significantly associated with the triage levels of the CHT and ATS (*p* < 0.001). The area under the receiver operating characteristic (AUROC) curve values of the CHT and ATS for predicting intensive care treatment were 0.845 (95% CI: 0.825–0.866) and 0.740 (95% CI: 0.715–0.765), respectively. The reliability and validity of the CHT and ATS were moderate, and both of them can be used to identify critical patients in emergency departments. It is necessary to further improve the triage system in terms of structure and content.

## 1. Introduction

With the increase in population growth, population aging, and hospital survival rates, boarding and crowding in emergency departments (EDs) has become a global problem [1–3]. However, patients' reasons for visiting the ED are varied: some because of the urgency of their health status itself and some because of their perceived structural environment and individual motivation [4]. Because of its urgency, indefiniteness, and complexity, emergency medicine faces serious challenges in preventing adverse events. When patients came to seek emergency aid, they first undergo triage. The first assessment of patients by triage nurses is a key factor in determining how the patient's ED experience will progress [4]. Many triage systems have been developed to help doctors and nurses make accurate decisions [5]. It is necessary to analyze the reliability and validity of the triage system to improve its accuracy in the future.

At present, four major emergency triage scales are used to determine the priority of care for a patient: the Australasian Triage Scale (ATS), the Canadian Triage and Acuity Scale (CTAS), the Manchester Triage System (MTS), and the Emergency Severity Index (ESI). Many scholars have studied these scales and have shown that their overall reliability is moderate [6–9]. However, differences have been revealed in the internal consistency of the system between nurses [7] and between nurses and doctors [8]. Some scholars have also compared and evaluated the reliability and validity of different triage scales, such as the CTAS and ESI, [9] the ESI and MTS, [10] and the MTS and ATS [11]. Few studies have examined the connection between the triage category and the length of stay or mortality rate. It has been reported that the ESI is associated with the length of stay or mortality rate [12] and inversely associated only with 6-month survival [13].

In China, the Ministry of Health announced “The guidelines for the classification of emergency patients (draft for solicitation)” in September 2011, which divided an ED into “three districts” (red, yellow, and green) and the patient's condition into “four levels” (acute and dangerous, severe acute, emergency, and nonemergency) [14]. These guidelines were simplified as “The Chinese four-level and three-district triage standard” (CHT) [14]. In China, only approximately half of hospitals use the formal scale, predominantly 4-tier (43%) or 5-tier (34%) scales [15]. So far, the implementation of the CHT in hospitals has rarely been reported [16]. Thus, few data are available that can be used to compare this standard with others. The earliest triage tool, the ATS, was developed in Australia and is widely used [6]. Although its use in China has not been reported, the ATS, like the CHT, was carried out by specifically trained and experienced registered nurses [17]. Therefore, we determined and compared the reliability and validity of the CHT and the ATS by analyzing their association with patient physiological status and clinical outcome.

## 2. Methods

### 2.1. Study Subjects

This observational study took place in a tertiary academic teaching hospital admitting more than 100 thousand emergency patients each year, which was conducted between January 2016 and January 2017. The hospital's adult ED is dedicated to handling patients older than 14 years. Those who died before hospital admission, refused treatment, or had incomplete data were excluded.

During the study period, all patients arriving at the ED were triaged by emergency nurses, who had at least three years of experience, working in the rescue area. All nurses were approached about participating in the study after being trained to use the CHT and ATS. A total of 12 nurses participated in the study. There were two nurses (an instructor nurse and an assistant nurse) per shift, who performed the triage duties for emergency patients; and the assignments were randomized by the head nurse. All patients included in the study were registered between 08:00 and 22:00 because there were two nurses on duty during this period. We collected data on 1585 patients according to the 0.01 accuracy of a simulation of power based on 1000 samples [18]. However, 1552 valid samples were ultimately obtained.

### 2.2. Triage Tools

#### 2.2.1. The Chinese Four-Level and Three-District Triage Standard (CHT)

According to the CHT [14], each patient was categorized into one of the four acuity levels. Each level defined the urgency of the condition: category 1 (dangerous and acute) = critically ill patients needing to immediately receive life-saving interventions; category 2 (severe acute) = the patient's condition is likely to deteriorate to grade 1 within a short time or may cause serious disability, and treatment should be arranged as soon as possible; category 3 (emergency) = no signs of life-threatening or serious disabilities within a short period, thus, necessitating attention within a certain period of time; and category 4 (nonemergency). Then, based on the acuity level, the nurse assigned the patient to one of the three treatment areas: the red zone, yellow zone, or green zone [14]. The emergency division of this research hospital was as follows: the red zone included the resuscitation room for category 1 and the rescue room for category 2, both of which were open intensive care units and in which patients were treated by the same group of emergency doctors and nurses; the yellow and green areas were emergency clinics for categories 3 and 4. In general, patients assigned to categories 3 and 4 were treated in chronological order. If the patient's condition changed during the waiting period or if the triage nurse considered it necessary, the patient was seen earlier or sent to the red zone immediately.

#### 2.2.2. The Australasian Triage Scale (ATS)

The ATS is a 5-level triage system that requires the user to select from a standardized set of patient statuses [19]. It is endorsed by the Australasian College for Emergency Medicine (ACEM). The ATS was translated into Chinese for ease of use in China using Brislin's translation model [20]. First, two bilingual nurses (one of whom was an emergency specialist nurse) translated the original scale into Chinese; then, the Chinese version was back-translated by a third bilingual expert, who did not see the original scale. This process was repeated to ensure that the final Chinese version of the ATS expressed the original intention. The triage assessment involves the presenting problem, the patient's general appearance, and pertinent physiological observations, comprising 79 indicative clinical descriptors. ED patients are allocated to categories 1 through 5 (1: immediate, 2: 10 minutes, 3: 30 minutes, 4: 60 minutes, and 5: 120 minutes) [19]. Any patient identified as ATS category 1 or 2 should be taken immediately into an appropriate assessment and treatment area.

### 2.3. Data Collection and Outcome Definitions

The CHT has already been used in our ED. Nurses applied the CHT and assessed the patient's general condition, respiratory and circulatory state, and complaint upon arrival to the triage area, and then classified the patient by means of the ATS, which only requires the most important information to be matched with the triage items to determine the triage level.

The first stage of the study involved a reliability analysis. During the first week, the instructing nurse and the assistant nurse used the two scales, the CHT and the ATS, to triage the same patient. Both nurses were blinded to each other's triage assessment and level assignment. During this process, 254 patients were assessed to compare the reliability of the CHT and ATS across different nurses. Next, one of the nurses continued to use the CHT and ATS to triage the same patient. A total of 1298 patients were assessed. The comparison of reliability of the ATS and CHT was based on the 254 samples from the triage performed by the instructing nurse during the first week plus the data from the additional 1298 patients. The doctor receiving time, disposal time, ED admission status, hospital admission rate (including deaths), and length of stay (LOS) were collected from the electronic hospital information systems. Patients who were transferred or signed out to return home were asked to confirm their outcomes via telephone at one month after discharge.

The definitions of the outcomes are as follows: waiting time = time from emergency registration to being seen by a doctor; treatment time = time from emergency registration to the administration of intervention measures, such as oxygen, fluids, medications, and physician health advice; length of stay = time from emergency registration to discharge from the hospital; hospitalization rate = the proportion of inpatients to the total number of patients at each triage level; and mortality rate = the proportion of deaths to the total number of patients at each triage level.

### 2.4. Statistical Analyses

Statistical analysis was performed using SPSS 21.0. First, the collected paperboard data were entered into the SPSS software and checked by two researchers. The degree of reliability between the CHT and the ATS was calculated using the weighted *k* scores. To facilitate statistical analysis, we merged ATS categories 3 and 4 into category 3 because ATS category 3 was defined as potentially life-threatening and situationally urgent, and category 4 was defined as potentially serious, situationally urgent, and significantly complex or severe, which is equivalent to CHT category 3. ATS category 4 in the figure and the table of this study corresponds to ATS category 5, which is equivalent to CHT category 4 (nonemergency). Then, a parametric analysis was performed after verifying the normal distribution of the continuous variables using the Kolmogorov–Smirnov test. All continuous variables had nonnormal distributions, and all were reported as 50th, 25th, and 75th percentiles. Statistical evaluation of the data was performed using the Kruskal–Wallis test, followed by pairwise comparisons. Categorical variables were reported as numbers and proportions. A chi-square test was used to analyze categorical variables, and the Bonferroni method was used to adjust the level of the test. Paired sample analysis was considered statistically significant if the adjusted *p* value was less than 0.05.

## 3. Results

### 3.1. Characteristics of the Study Subjects

A total of 1585 patients were triaged. Of these, eleven cases involved prehospital death, ten cases involved rejection of treatment, and twelve cases involved incomplete data, leaving 1552 patients for analysis, of which 56.96% were male and 43.04% were female. The patient age ranged from 14 to 99 years, with a median (Q25, Q75) age of 58.0 (44.0, 69.0) years. There were 1137 internal medicine cases, 133 surgical cases, 219 neurology cases, 31 neurosurgical cases, and 32 others. Forty-four patients had died at follow-up, when 150 follow-up calls were made one month after discharge.

### 3.2. Reliability Comparison of the Triage Criteria

Of the 12 triage nurses participating in the study, seven were intermediate nurses (two with more than 20 years of emergency experience) and five were junior nurses in terms of professional title. There was no difference between the two groups (the instructor nurses and the assistant nurses) in the distribution of professional titles. The reliability analysis of the same triage tool between the two groups of nurses was based on the data from the 254 initial patients. The total consistency *k* scores was 0.686 (95% CI 0.608–0.757) between the two groups of nurses for the CHT and 0.731 (95% CI 0.663–0.790) for the ATS. The comparison of reliability between the CHT and ATS originated from the data collected when the same nurse applied these two triage tools to allocate the same patient. The overall consistency *k* scores was 0.654 (95% CI 0.622–0.689) and 0.630 (95% CI 0.594–0.669) when ATS grades 3 and 4 were combined. The results are shown in Table 1.

### 3.3. Comparison of the Validity of the Triage Criteria

#### 3.3.1. Distribution of Triage Categories for the Triage Criteria

**Figure 1:**
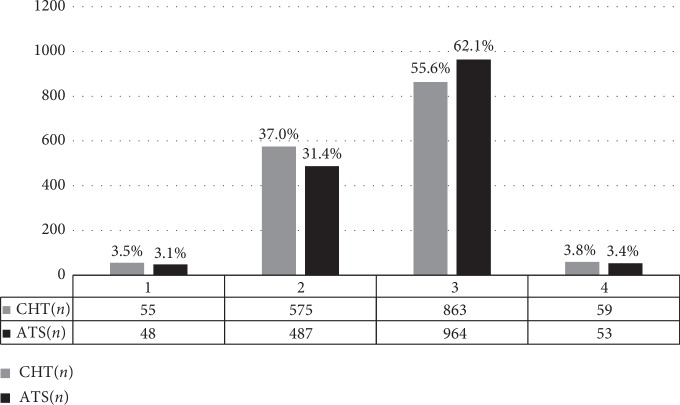
Distribution of different triage categories for the CHT and ATS (*n* = 1552). Note: ATS categories 3 and 4 were merged into category 3, which is equivalent to CHT category 3 (emergency); ATS category 4 in this figure corresponds to ATS category 5, which is equivalent to CHT category 4 (nonemergency).

The number of patients in each category of the triage criteria is shown in Figure 1. There were differences in distribution between the CHT group and ATS group (*p* < 0.001). When the two triages were compared as four-level triages, the distribution of triage categories was shown to be significantly different (*p*=0.003). Except for categories 1 and 4, the intercomparisons between the other groups were statistically significant, *p* < 0.05.

#### 3.3.2. Relationships of Triage Category with Physiological Indicators and Outcomes

Physiological indicators such as temperature, respiration, pulse, and blood oxygen saturation were strongly associated with the CHT and the ATS triage categories (*p* < 0.001). After adjusting for multiple comparisons, the respiratory rates of patients in category 1 and category 2 were significantly higher than those of patients in categories 3 and 4 (*p* < 0.05), and the pulse rate of patients in category 1 was significantly higher than that of patients in the other categories (*p* < 0.05). The blood oxygen saturation value of category 1 and category 2 was significantly lower than that of category 3 and category 4 patients (*p* < 0.05).

Similar results were found for the following triage outcomes: waiting time, treatment time, emergency disposition, hospitalization rate, and mortality (*p* < 0.001). The hospitalization rates of patients classified as category 2 according to both the CHT and ATS were significantly higher than those of patients in categories 3 and 4 (*p* < 0.05), but the length of stay and mortality were significantly higher for category 1 patients (*p* < 0.05). The results are shown in Table 2.

#### 3.3.3. Performance of Emergency Triage

**Figure 2:**
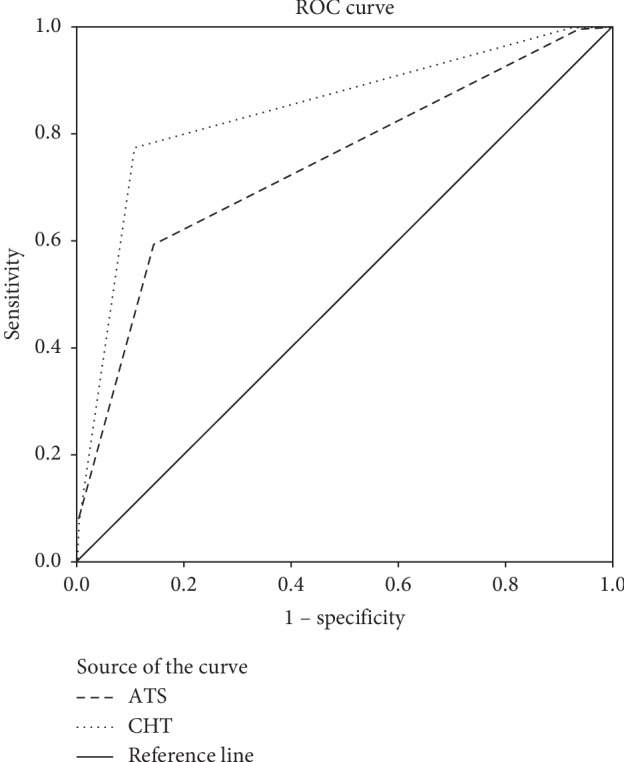
The area under the receiver operating characteristic (AUROC) curves to predict intensive care (*n* = 1552).

In Figure 2, the areas under the receiver operating characteristic (AUROC) curves of the CHT and the ATS for predicting intensive care are shown. The AUROC values were 0.845 (95% CI: 0.825–0.866) and 0.740 (95% CI: 0.715–0.765), respectively.

## 4. Discussion

In this study, the reliability of the CHT was similar to that of the ATS. Overall, the CHT triage validity was consistent with that of the ATS. A lower triage category number was related to deterioration of health status, a faster response speed, increased hospitalization time, a higher hospitalization rate, and a higher mortality rate.

According to the Fleiss grade [21] and using 0.65–0.70 as an acceptable range, it was suggested that the CHT and ATS had good reliability. The internal consistency *k* scores of the ATS was 0.731, which was consistent with the *k* scores of 0.64∼0.82 in the ATS scenario evaluation [22, 23] and slightly higher than that in the CHT *k* score of 0.686 in this study. Nurses tended to combine the CHT with clinical experience because the CHT is a comprehensive assessment based on vital signs rather than precise digital evaluation. Nevertheless, the ATS has corresponding items, the nurses matched the patient's condition with each item. However, the AUROC curve value of the CHT 0.845 was slightly higher than the ATS value of 0.740, as shown in Figure 2. In this study, two nurses independently triaged the same patient on the spot at the same time, and the results fully reflected the actual situation.

Based on the analysis of the population distribution, CHT and ATS category 2 and category 3 patients accounted for 30% to 40% and 50% to 60% of patients, respectively, indicating that relatively large numbers of potential crisis or emergency patients visited the ED of this tertiary hospital. This provincial general hospital is one of the main acceptance points for the transfer of critically ill patients from regional hospitals. Most of the patients were middle aged or elderly, with an average age of 58.0 (44.0, 69.0) years. Some data have shown that the rate of emergency visits has been increasing every year, especially for the elderly and for patients in triage categories 2 and 3 [2, 24]. This finding reflects not only the population increasing and aging but also the urgency and complexity of patients' conditions; such patients require more observation and treatment than can be provided in outpatient visits.

In the past, many studies evaluated triage validity in terms of hospitalization rate, mortality rate, medical resources, and medical expenses [10, 11, 25]. This study also evaluated physiological indicators, hospitalization rate, mortality rate, and other parameters. Respiration, pulse rate, and oxygen saturation were closely related to different categories, but there was no significant correlation in blood pressure. Earlier studies also showed that the worsening of vital signs, such as awareness, breathing, and oxygen saturation, increased the probabilities of ICU stay and death [26, 27]. Some studies have reported that systolic blood pressure is closely related to disease severity. Hypotension (<90 mmHg) is closely associated with hospitalization and death [28]; conversely, hypertension has a protective effect [27]. No difference in blood pressure by triage category was found in our study, possibly because no stratification analysis was performed.

The waiting time and treatment time were used to further evaluate the effect of medical staff complying with the systems of triage criteria. The waiting times of patients in category 1 and category 2 were shorter than those of patients in category 3 and category 4. When a patient was assigned to category 1, he or she was immediately sent to the resuscitation room by the triage nurses. At the same time, the doctors and nurses were informed to launch interventions such as respiratory support and circulatory support. Some category 2 patients were directly sent to the rescue room by nurses, and some were admitted to the intensive care unit after being evaluated by the doctors in the diagnosis area. The waiting time of category 2 patients (2.0 min) was very short, almost equal to that of category 1 patients (1.0 min), which indicated that the triage nurses and doctors paid close attention to these patients and prioritized their care. In addition, it may also be that because triage category 1 and 2 patients were immediately moved to the red zone, where a doctor would presumably see the patient soon, the difference in waiting time was not obvious. However, the treatment time for category 1 patients (1.0 min) was significantly shorter than for category 2 patients (8.0 min), which reflected the rapid response to patients with life-threatening conditions. The category 1 patients were critical and often deteriorated rapidly to death before hospitalization. This factor may have led to a higher mortality rate but a lower hospitalization rate in category 1 relative to category 2. No patient in category 4 was found to have died.

Our study confirmed that the CHT and the ATS were significantly correlated with the patients' condition, emergency treatment, and clinical outcomes, which can distinguish the severity of emergency patients. The AUROC curve value of the CHT was slightly higher, at 0.845 (95% CI: 0.825–0.866), than that of the ATS, at 0.740 (95% CI: 0.715–0.765), which might also be explained by the triage way of CHT. Based on the triage level of patients, the CHT triage standard stipulates the patients' treatment area. On the one hand, this way helps shorten the time of obtaining treatment for critical patients, and on the other hand, it can enhance nurses' risk awareness and send critically ill patients to the open ICUs (the resuscitation room or rescue room) to avoid delayed treatment.

## 5. Limitations

This study has several limitations. First, the data were obtained from a single setting. In the course of triage, some patients were transferred from other hospitals and had usually undergone clinical assessments, accompanied by auxiliary examinations and even a definitive final diagnosis, which was beneficial for the triage. The information collected in this study was based on paperboard surveys, and there may be typical sample cases. Hence, future studies with larger network samples are needed to better interpret our findings. Second, although this study was based on the real scenarios, this method could not be completely blinded because the assistant nurse could see which zone the patient was sent to by the instructing nurse and could adjust his or her CTS and ATS triage level accordingly for the subsequent patients. The presence of this “learning curve” could have contributed to the higher ATS consistency of 0.731 in this study than that of 0.64 [22] obtained from simulated triage scenarios based on actual ED patients. It also happened in the CHT consistency results. The accuracy of the triage was also influenced by nurses' knowledge and experience, the external environment, the institutional level, and bed availability. Finally, this research was conducted in an adult ED, so the results cannot be applied to pediatric patients.

## 6. Conclusions

This study compared the reliability and the validity of the CHT and the ATS. The reliability levels of the CHT and the ATS were considered moderate. Both systems can be used to identify critical patients in EDs. In order to improve the reliability and validity of emergency triage, it is necessary to further study the triage system in terms of structure and content.

## Figures and Tables

**Table 1:** Interrater reliability for the CHT and ATS.

Triage system	*k* scores	95% CI
CHT by instructor group with CHT by assistant group (*n* = 254)	0.686	0.608–0.757
ATS by instructor group with ATS by assistant group (*n* = 254)	0.731	0.663–0.790
CHT with ATS (*n* = 1552)	0.654	0.622–0.689
CHT with ATS (combination of level 3 and level 4, *n* = 1552)	0.630	0.594–0.669

*Note*. CHT, three-district and four-level triage standards of the Chinese Ministry of Health; ATS, Australasian Triage Scale.

**Table 2:** Association of the triage category with physiological indexes and outcomes (*n* = 1552).

Variable	Triage system	Triage category				*P*
		Level 1	Level 2	Level 3	Level 4	
Age (years, $)	CHT	57.0 (44.0, 69.0)	60.5 (49.0, 71.0)	55.5 (45.0, 68.0)^*b∗∗*^	57.5 (30.5, 70.0)^*b∗∗*^	<0.001
	ATS	59.0 (45.7, 70.0)	60.0 (49.0, 70.0)	58.0 (44.0, 69.0)^*b∗∗*^	64.0 (51.7, 68.0)^*b∗*^	<0.001

Temperature (°C, $)	CHT	36.5 (36.1, 37.7)	36.5 (36.4, 36.8)	36.7 (36.5, 36.8)^*b∗∗*^	36.6 (36.5, 36.8)	<0.001
	ATS	36.7 (36.2, 37.3)	36.5 (36.4, 36.8)	36.7 (36.5, 36.8)^*b∗∗*^	36.5 (36.4, 36.9)^*c∗*^	<0.001

Respiration (times/min, $)	CHT	21.0 (20.0, 28.0)	20.0 (20.0, 24.00)	20.0 (20.0, 20.0)^*a∗∗b∗∗*^	20.0 (20.0, 20.0)^*a∗∗b∗∗*^	<0.001
	ATS	20.5 (20.0, 26.5)	20.0 (20.0, 22.0)	20.0 (20.0, 22.0)^*a∗∗b∗∗*^	20.0 (20.0, 20.0)^*a∗∗b∗∗c∗∗*^	<0.001

Pulse (times/min, $)	CHT	118.0 (90.0, 134.0)	90.0 (79.0, 108.8)^*a∗∗*^	87.0 (79.0, 103.5)^*a∗∗*^	87.5 (84.2, 101.5)^*a∗∗*^	<0.001
	ATS	107.5 (90.0, 126.5)	87.0 (78.0, 107.0)^*a∗∗*^	90.0 (80.0, 105.0)^*a∗∗*^	73.5 (71.7, 90.7)^*a∗∗*^	0.001

Systolic blood pressure (mmHg, $)	CHT	135.0 (120.0, 159.0)	131.0 (110.0, 154.0)	124.0 (111.0, 150.0)	127.5 (112.0, 141.5)	-
	ATS	148.0 (129.7, 169.5)	133.0 (112.0, 159.0)	124.0 (110.0, 148.0)	150.0 (108.0, 168.0)	0.042

Diastolic blood pressure (mmHg, $)	CHT	83.0 (70.0, 99.0)	77.0 (66.0, 89.0)	75.0 (67.0, 86.0)	69.5 (63.0, 84.5)	-
	ATS	81.0 (70.0, 102.0)	79.0 (68.0, 90.0)	74.5 (66.0, 86.0)	78.0 (72.5, 88.0)	0.036

Blood oxygen saturation (%, $)	CHT	92.0 (80.0, 98.00)	96.0 (91.0, 98.0)^*a∗∗*^	97.0 (95.0, 98.0)^*a∗∗b∗∗*^	98.0 (97.2, 98.7)^*a∗∗b∗∗*^	<0.001
	ATS	91.0 (78.0, 96.5)	97.0 (91.0, 98.0)^*a∗∗*^	97.0 (95.0, 98.0)^*a∗∗b∗∗*^	97.5 (96.2, 99.0)^*a∗∗b∗∗*^	<0.001

Waiting time (min, $)	CHT	1.0 (1.0, 4.0)	2.0 (1.0, 5.0)	9.5 (2.0, 21.7)^*a∗∗b∗∗*^	18.0 (10.0, 38.2)^*a∗∗b∗∗*^	<0.001
	ATS	1.0 (1.0, 2.0)	2.0 (1.0, 5.0)^*a∗∗*^	8.0 (2.0, 20.0)^*a∗∗b∗∗*^	17.5 (4.3, 34.5)^*a∗∗b∗c∗*^	<0.001

Treatment time (min, $)	CHT	1.0 (1.0, 2.0)	8.0 (2.0, 28.7)^*a∗∗*^	48.5 (25.0, 95.0)^*a∗∗b∗∗*^	134.5 (85.5, 184.0)^*a∗∗b∗∗c∗*^	<0.001
	ATS	1.0 (1.0, 4.5)	12.0 (2.0, 44.0)^*a∗∗*^	35.0 (15.0, 78.3)^*a∗∗b∗∗*^	97.5 (83.7, 138.7)^*a∗∗b∗∗c∗∗*^	<0.001

Length of stay (days, $)	CHT	18.0 (11.5, 28.0)	12.0 (9.0, 18.0)^*a∗*^	12.0 (8.0, 16.0)^*a∗*^	10.5 (9.0, 15.7)^*a∗*^	0.019
	ATS	18.0 (11.3, 28.0)	12.0 (8.5, 19.0)^*a∗*^	12.0 (8.7, 16.0)^*a∗*^	14.0 (6.8, 21.3)	0.023

Hospitalization rate (*n*, %)	CHT	35 (63.6)	416 (72.3)	418 (48.4)^*b∗*^	12 (20.3)^*a∗b∗c∗*^	<0.001
	ATS	32 (66.7)	349 (71.7)	494 (51.2)^*b∗*^	6 (11.3)^*a∗b∗c∗*^	<0.001

Total mortality rate (*n*, %)	CHT	18 (32.7)	109 (19.0)	38 (4.4)^*a∗b∗*^	-	<0.001
	ATS	19 (39.6)	87 (17.9)^*a∗*^	57 (6.1)^*a∗b∗*^	-	<0.001

Emergency mortality rate (*n*, %)	CHT	12 (21.8)	46 (8.0)^*a∗*^	9 (1.0)^*a∗b∗*^		<0.001
	ATS	9 (18.8)	38 (7.8)	20 (2.1)^*a∗b∗*^		<0.001

Disposition (*n*, %)	CHT					<0.001
	ICU	51 (92.7)	491 (85.4)	158 (18.3)^*a∗b∗*^	-	
	Observation	3 (5.5)	73 (12.7)	434 (50.3)^*a∗b∗*^	24 (40.7)^*a∗b∗*^	
	Discharge	1 (1.8)	8 (1.4)	218 (25.3)^*a∗b∗*^	27 (45.8)^*a∗b∗c∗*^	
	Outpatient	—	3 (0.5)	53 (6.1)^*b∗*^	8 (13.6)^*b∗*^	
	ATS					<0.001
	ICU	47 (97.9)	367 (75.4)^*a∗*^	284 (29.5)^*a∗b∗*^	2 (3.8)^*a∗b∗c∗*^	
	Observation	1 (2.1)	99 (20.3)^*a∗*^	422 (43.8)^*a∗b∗*^	12 (22.6)^*a∗b∗c∗*^	
	Discharge		16 (3.3)	204 (21.2)^*b∗*^	34 (64.2)^*b∗c∗*^	
	Outpatient		5 (1.0)	54 (5.6)^*b∗*^	5 (9.4)^*b∗*^	

*Note*. *a∗*, *p* < 0.05 compared with level 1; *a∗∗*, *p* < 0.01 compared with level 1; *b∗*, *p* < 0.05 compared with level 2, *b∗∗*, *p* < 0.01 compared with level 2; *c∗*, *p* < 0.05, compared with level 3, *c∗∗*, *p* < 0.01 compared with level 3. CHT, three-district and four-level triage standards of the Chinese Ministry of Health; ATS, Australasian Triage Scale; $, median (Q25, Q75).

## Data Availability

The data used to support the findings of this study are restricted by the Ethics Committee of the Second Xiangya Hospital, China (2016/S014).
